# Postoperative hypothermia and surgical site infection following peritoneal insufflation with warm, humidified carbon dioxide during laparoscopic colorectal surgery: a cohort study with cost-effectiveness analysis

**DOI:** 10.1007/s00464-016-5195-0

**Published:** 2016-10-12

**Authors:** Sam E. Mason, James M. Kinross, Jane Hendricks, Thanjakumar H. Arulampalam

**Affiliations:** 1 ICENI Centre, Colchester Hospital, Turner Road, Colchester, Essex CO4 5JL UK; 2 Department of Surgery and Cancer, Imperial College London, St Mary’s Hospital, London, UK

**Keywords:** Postoperative hypothermia, Surgical site infection, Carbon dioxide conditioning

## Abstract

**Background:**

Surgical Site Infection (SSI) occurs in 9 % of laparoscopic colorectal surgery. Warming and humidifying carbon dioxide (CO_2_) used for peritoneal insufflation may protect against SSI by avoiding postoperative hypothermia (itself a risk factor for SSI). This study aimed to assess the impact of CO_2_ conditioning on postoperative hypothermia and SSI and to perform a cost-effectiveness analysis.

**Methods:**

A retrospective cohort study of patients undergoing elective laparoscopic colorectal resection was performed at a single UK specialist centre. The control group (*n* = 123) received peritoneal insufflation with room temperature, dry CO_2_, whereas the intervention group (*n* = 123) received warm, humidified CO_2_ (using HumiGard™, Fisher & Paykel Healthcare). The outcomes were postoperative hypothermia, SSI and costs. Multivariate analysis was performed.

**Results:**

A total of 246 patients were included in the study. The mean age was 68 (20–87) and mean BMI 28 (15–51). The primary diagnosis was cancer (*n* = 173), and there were no baseline differences between the groups. CO_2_ conditioning significantly decreased the incidence of postoperative hypothermia (odds ratio 0.10, 95 % CI 0.04–0.23), with hypothermic patients found to be at increased risk of SSI (odds ratio 4.0, 95 % CI 1.25–12.9). Use of conditioned CO_2_ significantly decreased the incidence of SSI by 66 % (*p* = 0.04). The intervention group incurred costs of £155 less per patient. The incremental cost-effectiveness ratio was negative.

**Conclusion:**

CO_2_ conditioning during laparoscopic colorectal surgery is a safe, feasible and a cost-effective intervention. It improves the quality of surgical care relating to SSI and postoperative hypothermia.

Surgical Site Infections (SSIs) have long been recognised as a barrier to the delivery of high-quality surgical care, representing up to 20 % of all healthcare-associated infections [1]. Surgery on the colon and rectum is responsible for the highest incidence of SSIs across all surgical specialties, likely owing to a multitude of factors including having a co-morbid population, relatively long procedure times and a high risk of bacterial contamination of the operative field. Non-emergent colorectal surgery has witnessed a transition over the past two decades from an open to laparoscopic approach to the abdominal cavity, which has seen a significant reduction in SSIs. Despite this intervention, elective colorectal SSI incidence remains at 8–9 %, a significant burden on healthcare institutions and patients alike [2, 3]. In order to limit the incidence and impact of SSIs, several regulatory bodies have published guidelines on the delivery of care in the perioperative period [1, 4]. Given the scale of colorectal surgery, a modest improvement in SSI incidence is likely to translate to a significantly reduced burden on institutions.

Maintenance of perioperative normothermia is now considered essential. This is based on findings that perioperative patient warming is independently protective in the development of SSIs [5, 6]. In order to perform effective and safe laparoscopic surgery, pneumo-peritoneum must be established. This is most commonly achieved with insufflation of carbon dioxide (CO_2_) from compressed steel cylinders, entering the peritoneal cavity at 21 °C with a relative humidity of 0 % [7]. Devices such as HumiGard (Fisher & Paykel Healthcare Ltd, Auckland, New Zealand) and Insuflow (Lexion Medical, St Paul, USA) therefore condition CO_2_ before delivery to the patient, raising its temperature to 37-40 degrees centigrade at relative humidity of >95 % (data from manufacturers). Prolonged peritoneal insufflation with cool (at a temperature <21 °C), dry CO_2_ in comparison with warm, humidified CO_2_ has been demonstrated to decrease core body temperature and increase the risk of perioperative hypothermia [7, 8].

The prevention of early postoperative hypothermia through CO_2_ conditioning may reduce the risk of developing an SSI; however, to date there is no evidence to support this hypothesis.

The aim of this retrospective cohort study was to evaluate the effect of warm, humidified CO_2_ on hypothermia and surgical site infections and to perform a cost-effectiveness analysis of its use.

## Materials and methods

### Study design and sample

A retrospective cohort study was conducted at a single specialist laparoscopic unit in the UK (Colchester University Hospital) between September 2012 and July 2014. Patients who underwent an elective laparoscopic resection of the colon, rectum or anus for malignant or benign disease were suitable for inclusion. Patients having elective laparoscopic reversal of Hartmann’s procedures were also included. Those undergoing emergency procedures or solely open procedures were excluded from the study.

No power calculation of sample size was conducted given the lack of literature regarding the impact of CO_2_ conditioning on hypothermia and SSI in elective colorectal surgery. The intervention group consisted of all patients who had elective laparoscopic colorectal surgery between July 2013 and July 2014. They received peritoneal insufflation with warm, humidified CO_2_, after installation of the HumiGard™ CO_2_ conditioning device (Fisher & Paykel Healthcare Ltd, Auckland, New Zealand). The control cohort was matched in size and consisted of a consecutive series of patients who underwent surgery from September 2012 to June 2013, immediately prior to the implementation of the device. They received peritoneal insufflation with standard dry, cool CO_2_ using a standardised protocol. Given the retrospective nature of this study, it was not necessary to seek research ethics committee approval or individual patient consent.

### Standardised patient care

Beyond the experimental intervention, both groups received identical pre-, peri- and postoperative care. The six consultant surgeons responsible for delivery of patient care were experienced laparoscopic specialists in colorectal surgery who did not change during the study period. Procedures were carried out either by the consultant or by a trainee under their supervision. All patients had negative preoperative screening cultures for Methicillin-resistant *Staphylococcus aureus*.

All patients underwent a standardised set-up in the operative suite, aided by the use of a preoperative checklist. Following induction of general anaesthetic, all patients received intravenous antibiotic prophylaxis in accordance with local guidelines, consisting of 1.2 g co-amoxiclav with a repeat dose administered if the operation was ongoing at 4 h. The operative position was at the discretion of the surgeon, with the Lloyd-Davis or supine used in the majority of cases. Standard-of-care intra-operative patient warming consisted of a forced-air warming blanket and the use of intravenous fluid warmers, both set-up for all patients and operated at the discretion of the anaesthetist.

Entry into the peritoneal cavity was routinely achieved with closed insertion of a blunt 5-mm metallic trocar into the flank. In cases of extensive previous abdominal surgery when blind entry was contraindicated, an open technique was used. Prior to July 2013, pneumo-peritoneum was established with insufflation of filtered, dry, cool CO_2_, harvested from a steel cylinder and delivered via a standard single-lumen tubing. The HumiGard™ CO_2_ conditioning device was installed in July 2013 and placed on the stack system beside the insufflator. It delivered warmed and humidified CO_2_ to the patient via a dual-lumen insulated tubing system, at a temperature of 37 degrees centigrade and humidity of greater than 98 % [9]. Flow rate and intra-abdominal pressure were controlled at the discretion of the operating surgeon. If an abdominal incision was necessary for specimen extraction, this was by midline laparotomy in all cases.

Postoperatively, all patients were admitted to a dedicated elective surgical ward or a high dependency suite based on clinical need, with at least twice daily dedicated rounds from an enhanced recovery surgical team. Administration of antimicrobial medications during this period was at the discretion of the consultant surgeon, and incision sites only underwent microbiological examination if a SSI was suspected. The enhanced recovery protocol including discharge criteria, wound care and duration of routine follow-up did not change during the study period.

### Outcome assessment

All patients expected to undergo laparoscopic elective colorectal surgery at Colchester Hospital are contemporaneously added to a departmental database, containing patient, operative and postoperative data. In particular with regard to the postoperative recovery, data are recorded including surgical complications, unplanned re-intervention, LOS and readmissions. From this database, patients were identified and data were extracted for analysis.

The primary outcome measure was the incidence of postoperative hypothermia. The secondary outcome measures were the incidence of SSI, LOS and performing a cost-effectiveness analysis.

Operative time was defined as the duration in minutes from initial surgical incision to the application of wound dressings. Body temperature was routinely measured tympanically on arrival to the post-anaesthetic recovery suite, with hypothermia defined as a temperature of less than 36 degrees centigrade. The measurement of temperature intra-operatively was not standardised and therefore could not be included in this analysis.

Surveillance for SSI was conducted by consultant surgeons, senior surgical nurses and infection control nurses, all trained in SSI identification. SSI was defined using objective clinical and microbiological criteria, in accordance with guidance from Public Health England [10]. In line with SSI definition, infections had to be identified within 30 postoperative days; therefore, any SSIs identified on patients within this time period were included. This included patients who had an SSI diagnosed on a readmission or at clinic follow-up.

### Statistical analysis

Patient data were anonymised and tabulated in Microsoft Excel^(c)^ 2010 (Microsoft Corporation, Redmond, Washington, USA). Parametric data were expressed as a mean ± standard deviation (SD) and dichotomous outcomes as raw number and percentage of total.

Statistical analyses were conducted using IBM SPSS Statistics (IBM Corp., New York). The cohorts were compared at baseline for similarity using Pearson’s [2] and Fisher’s exact tests for dichotomous data (for greater and less than five available data points, respectively). Continuous data were analysed with a two-tailed independent Student’s *t* test. Predictive factors for development of hypothermia and SSI were estimated using multivariate analyses. For all statistical tests, a ‘*p*’ value of <0.05 indicated statistical significance. A subgroup analysis was performed including all patients who underwent resection of the left colon or rectum with intra-abdominal anastomosis for the management of cancer.

A cost-effectiveness analysis was conducted with regard to implementation of the CO_2_ conditioning device. The National Institute of Health and Care Excellence has published costs associated with SSIs, based on which an economic model was created [11]. Conservative estimates were first generated for minimum cost of an SSI (£500), most likely cost (£2 000) and maximum cost (£10 000). These were used to construct a PERT distribution, and following application of the central limit theorem, mean SSI cost was estimated at £3083 with a standard deviation of £1 597. Equipment expenditure was £75 and £10 in the intervention and control groups, respectively. Using these data, the incremental cost-effectiveness ratio (ICER) was calculated and defined as the incremental expenditure required to avoid one SSI.

## Results

A total of 276 patients were scheduled for laparoscopic colorectal surgery during the study period, with 30 excluded from this study as they underwent a solely open approach. Data of 123 patients in the control group and 123 patients in the intervention group underwent analysis.

There was no significant difference in baseline patient characteristics between the two groups for age, gender, body mass index, smoking status and diabetes mellitus (Table 1). The mean operation time was 213 min (range 29–690). The most common procedures were anterior resection, right hemicolectomy and sigmoid colectomy. There was no difference between the groups in terms of operation performed, duration of surgery, conversion rate and postoperative admission to the intensive care unit; however, a greater proportion of patients in the control group underwent surgery for malignancy.

**Table 1:** Patient and operative characteristics

Patient characteristic	Control (*n* = 123)	Intervention (*n* = 123)	*p* value
Mean age, years (range)	65 (20–87)	67 (23-86)	0.21^@^
Gender (M/F)	67:56	57:66	0.20*
Mean BMI ± SD (range)	28.2 ± 5.7 (15–45)	27.3 ± 5.2 (17–51)	0.24^@^
Smokers (%)	10.6	11.4	0.84*
Diabetes mellitus (%)	6.5	7.3	0.80*
Mean operation time ± SD, minutes	214 ± 91	213 ± 92	0.89^@^
Operation performed (*n*)			
Anterior resection	32	31	0.88*
Right hemicolectomy	31	31	1.00*
Sigmoid colectomy	21	20	0.86*
APR	9	7	0.61*
Left hemicolectomy	6	3	0.34^+^
Reversal of Hartmann’s	8	6	0.58*
Ileocaecal resection	4	2	0.45^+^
Other	12	23	
Conversion rate (%)	10.6	12.2	0.69*
Postoperative admission to ITU (%)	8.9	11.4	0.53*
Primary diagnosis (*n*)			
Malignant	94	79	0.04*
Diverticulitis	12	16	0.42*
Crohn’s disease	9	4	0.25^+^
Ulcerative colitis	1	6	0.12^+^
Other	7	18	

* Chi-squared test

^+^Fisher’s exact test

^@^Student’s t test

### Postoperative hypothermia

The incidence of postoperative hypothermia on arrival at the recovery suite was 57 and 13 % for the control and intervention groups, respectively (*p* ≤ 0.001). Multivariate analysis demonstrates that use of the CO_2_ conditioning device was the only variable to significantly modify the risk of the hypothermia, with an OR 0.10 (95 % CI 0.04–0.23, *p* < 0.001) (Table 2).

**Table 2:** Multivariate analysis of risk factors for development of postoperative hypothermia

Factor	Effect size (95 % CI)	*p* value
Diabetes mellitus	0.51 (0.11–2.27)	0.38
Male gender	1.67 (0.74–3.70)	0.22
Age (years)	1.01 (0.98–1.05)	0.50
BMI	0.96 (0.88–1.05)	0.33
Surgery for cancer	1.43 (0.49–4.17)	0.51
Operation time (minutes)	1.00 (0.99–1.00)	0.34
Conversion to open approach	2.94 (0.85–10.0)	0.09
Use of conditioned CO_2_	0.10 (0.04–0.23)	<0.001

*BMI* body mass index, *CO*_*2*_ carbon dioxide, *CI* confidence interval

### Surgical site infection

**Fig. 1:**
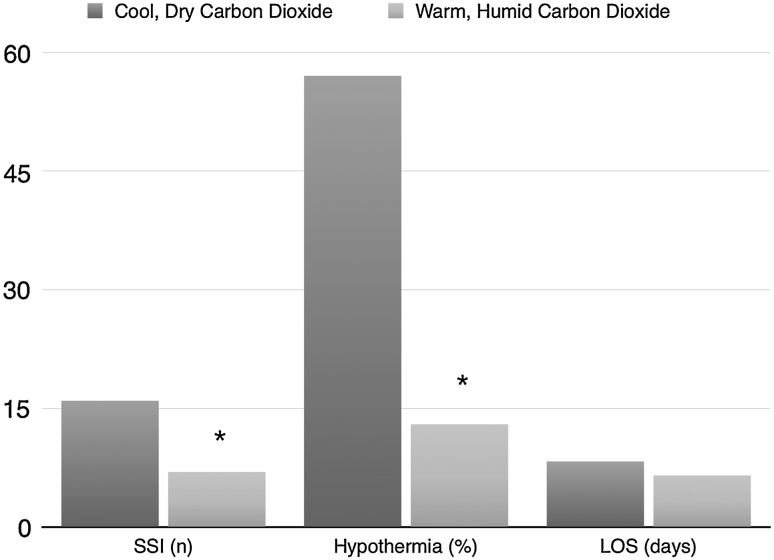
Effect of carbon dioxide conditioning on clinical outcomes. *Asterisk* denotes statistical significance

The overall incidence of SSI was 9.3 % (*n* = 23). Sixteen patients in the control group (13.0 %) and seven in the intervention group (5.7 %) developed an SSI (Fig. 1). There was no difference between the groups in the type of SSI identified. Each cohort experienced two organ space infections, one of which in each group was associated with anastomotic leak. Multivariate analysis demonstrates that each additional BMI point, male gender and conversion to open surgery are significant risk factors for development of an SSI, with a cumulative OR of 19.4  (Table 3). Use of the CO_2_ conditioning device significantly decreased the risk of SSI with OR 0.34 (*p* = 0.04). Postoperative hypothermia was not included in the multivariate analysis given the aim of the CO_2_ conditioner to modify this variable. However, it was noted that hypothermic patients had a significantly increased risk of developing an SSI with an OR of 4.0 (95 % CI 1.25–12.9, *p* = 0.02).

**Table 3:** Multivariate analysis of risk factors for developing SSI

Variable	Effect size (95 % CI)	*p* value
Male gender	3.42 (1.12–10.4)	0.03
Age (years)	1.01 (0.96–1.05)	0.82
BMI	1.09 (1.00–1.19)	0.04
Active smoker	1.70 (0.40–7.25)	0.47
Diabetes	1.36 (0.20–9.11)	0.76
Surgery for cancer	0.53 (0.17–1.80)	0.32
Operation time (minutes)	1.00 (1.00–1.01)	0.80
Conversion to open approach	5.21 (1.75–15.63)	0.003
Use of CO_2_ conditioner	0.34 (0.12–0.95)	0.04

*BMI* body mass index, *CO*_*2*_ carbon dioxide, *CI* confidence interval

### Length of Stay

The LOS ranged from 1 to 88 days, with a median of 5 days. Patients who developed an SSI had a median LOS 5 days longer (*p* = 0.002) and were significantly more likely to be readmitted to hospital within 30 days with an OR of 3.6 (95 % CI 1.27–10.1, *p* = 0.01). Patients who received cool, dry CO_2_ had an increased LOS in comparison with those receiving conditioned CO_2_ (Fig. 1); however, this was not statistically significant (8.3 vs. 6.4 days, *p* = 0.11).

### Subgroup analysis

The subgroup analysis consisted of 91 patients who underwent left-sided colon or rectum resection with primary anastomosis for cancer. In this group, use of the CO_2_ conditioning device continues to protect against hypothermia; however, male gender and conversion to open approach now significantly increase the risk of hypothermia (data not shown). In the consideration of SSI, BMI and conversion to open approach remained significant risk factors, with male gender and increased age risk factors that did not demonstrate statistical significance (*p* = 0.10 and 0.06, respectively, data not shown). The use of the CO_2_ conditioning device was no longer protective (*p* = 0.22).

### Cost-effectiveness analysis

The cost incurred relating to SSIs was estimated at £48 093 and £21 033 for the control and intervention cohorts, respectively, which reflected a cost burden per patient of £391 and £171. Once equipment costs were considered, £155 was saved on average per patient with the use of the conditioning device. The incremental cost-effectiveness ratio was negative.

## Discussion

This retrospective analysis provides evidence for the benefits of using humidified, warm CO_2_ *versus* dry, cool CO_2_ for peritoneal insufflation during elective laparoscopic colorectal surgery.

The primary outcome was the development of early postoperative hypothermia, a common finding in patients undergoing general anaesthetic [12]. This study demonstrated that use of warm, humidified CO_2_ for peritoneal insufflation significantly decreased the incidence of postoperative hypothermia and furthermore, that postoperatively hypothermic patients were at increased risk of SSI. The relationship between CO_2_ conditioning and postoperative hypothermia has been described [7], with the heat loss likely as a result of convection (insufflation gas directly warmed by the body and then removed) and evaporation (of intra-abdominal extra-cellular fluid). Our finding that postoperatively hypothermic patients were at increased risk of SSI has been shown across a variety of surgical specialities, including colorectal, trauma and vascular surgery [5, 13, 14].

The secondary outcome in this study was the incidence of SSI, which at 9.3 % is in keeping with previous case series in similar laparoscopic colorectal patients [15]. Warming and humidifying CO_2_ significantly decreased the risk of developing an SSI (13.0 vs. 5.7 %), readmission rates and length of hospital stay. The improvement in these objective clinical outcomes demonstrates delivery of a higher quality of surgical care, benefiting both our patients in addition to our institution.

By its very nature, surgical intervention provides a unique opportunity for the development of soft tissue infection, given the contamination of sterile tissues, interruption of innate protective barriers and the stimulation of a systemic stress response. Several mechanisms have been proposed to explain why hypothermic patients are at an increased risk of SSI. Hypothermia results in subcutaneous vasoconstriction, with decreased oxygen tension in this layer at a wound site shown to increase SSI risk [16, 17]. Hypothermia has also been shown in vitro to have a detrimental effect on the host’s ability to mount an immune response, decreasing the ability of leucocytes to migrate, produce antibodies and phagocytose [18]. It is also possible that CO_2_ conditioning protects against SSI via another mechanism, such as by reducing the extent of peritoneal dessication [19]. If any of the above mechanisms are the dominant factor in the SSI protection that CO_2_ conditioning affords, it may be reflected in the specific type of SSI the patient suffers. No difference between incisional and organ space infections was seen between the groups in this study; however, given the small number of SSIs, it is likely the sample sizes are underpowered to detect if CO_2_ conditioning protects against a specific type of SSI.

Despite inadequately understanding the mechanisms behind SSI development, the relationship between hypothermia and SSIs is sufficiently strong that Public Health England recommends surgical departments make interventions to avoid perioperative hypothermia, with audits of compliance. This study supports the practice of auditing and improving this surrogate outcome, showing that postoperative hypothermia was a risk factor for development of an SSI in this patient group. This is the first study to assess CO_2_ conditioning on the incidence of SSIs, and although our findings in conjunction with the literature imply perioperative hypothermia is the aetiology, the mechanism by which this is the case remains unclear.

Implementation of the CO_2_ conditioning device was feasible, requiring little space in the operative theatre, and a set-up time negligibly increased by filling a reservoir with sterile water. This study demonstrated no clinical adverse effects or operative suite issues that could be attributed to the device.

There appears to be a financial benefit to the institution by using CO_2_ conditioning equipment. The cost-effectiveness analysis demonstrates that despite deployment of this device being £65 more expensive per patient, the intervention is dominant over the control in practice by decreasing both SSI incidence and overall institution costs. This is reflected in the negative ICER. The negative value denotes that for every SSI avoided, the institution appears to save money. These savings are likely to relate to decreases in hospital stay, administration of antimicrobial medications and further operative intervention related to an SSI. Costs within this study were estimated based on figures published by the National Institute for Health and Care Excellence, due to the inability to gather patient level cost data locally. Despite potential bias associated with cost estimation, the authors consider the model to be conservative, likely underestimating the true cost given the increased length of stay and readmission rates seen during the study.

This is a novel technology which addresses several priorities in healthcare delivery. The National Health Service of the UK is currently under significant financial and safety pressures, with continued emphasis on making interventions to improve the quality of surgical care [20]. Implementation of this device appears to be such an intervention, where improvement can be detailed in both clinical and health economic outcomes. The economic benefits of protecting against hypothermia and SSI extend beyond avoiding the additional costs of managing these conditions, but also opportunity costs across a system with fixed resources. Clinical outcomes affect both the institution and the patient. This includes the avoidance of sepsis, which given its mortality implications in an ageing population and concerns over antibiotic resistance is of increasing importance in the surgical patient [21].

In order to interpret the findings of this study, its limitations must be considered. Given its retrospective nature, this study is at risk of systematic bias and in particular of making a type I error. As such, this study design would not have been able to identify if the decrease in SSI incidence over the study period was due to a confounding factor independent of the intervention. Such factors would be in relation to aspects of patient care that could not be standardised retrospectively, or where data could not be collected for inclusion in statistical analysis. This includes how anaesthetists chose to operate the other equipment for intraoperative warming and that the prescription of antimicrobials was at the discretion of consultant surgeons. The risk of bias from these factors is considered low given that the senior surgical and anaesthetic teams remained unchanged over the study period. In addition, antimicrobials were prescribed in close collaboration with microbiological physicians with unchanged local protocols. In future prospective studies, these factors, including operative factors such as blood loss, should be compared between groups. Selection bias was minimised by using consecutive patients to create the cohorts of interest, resulting in no differences in baseline characteristics between the groups. The risk of observation and recall biases was low. The outcome assessors were blinded to the intervention and collected data contemporaneously, before this study had been designed. Additionally, they were adequately trained in SSI detection. Despite the apparent low risk of systematic bias, it will be necessary to confirm these findings in a prospective cohort study, using the present data to ensure it is of adequate power.

## Conclusion

This retrospective cohort study demonstrates a likely benefit to the use of warm, humidified CO_2_ for peritoneal insufflation during elective colorectal surgery with regard to SSIs, postoperative hypothermia and length of stay. The intervention was found to be safe, feasible and cost effective. A prospective cohort study will be needed to confirm these findings.
